# Screening for Biofilm-Stimulating Factors in the Freshwater Planctomycete *Planctopirus limnophila* to Improve Sessile Growth in a Chemically Defined Medium

**DOI:** 10.3390/microorganisms10040801

**Published:** 2022-04-12

**Authors:** Oscar Kruppa, Peter Czermak

**Affiliations:** 1 Institute of Bioprocess Engineering and Pharmaceutical Technology, University of Applied Sciences Mittelhessen, 35390 Giessen, Germany; oscar.kruppa@lse.thm.de; 2 Faculty of Biology and Chemistry, Justus-Liebig University of Giessen, 35390 Giessen, Germany

**Keywords:** Planctomycetes, *Planctopirus limnophila*, chemically defined medium, fixed-bed cultivation, biofilm formation, MTT assay, crystal violet staining, metal ions, C:N ratio, oxidative stress

## Abstract

Planctomycetes such as *Planctopirus limnophila* offer a promising source of bioactive molecules, particularly when they switch from planktonic to sessile growth, but little is known about the corresponding biosynthetic gene clusters and how they are activated. We therefore screened for factors that promote sessile growth and biofilm formation to enable the cultivation of *P. limnophila* in a fixed-bed reactor. We carried out screening in microtiter plates focusing on biofilm formation and changes in optical density in response to various C:N ratios, metal ions, and oxidative stress. We used MTT assays and crystal violet staining to quantify biofilm formation. Positive factors were then validated in a fixed-bed bioreactor. The initial screen showed that D1ASO medium supplemented with NH_4_Cl to achieve a C:N ratio of 5.7:1, as well as 50 µM FeSO_4_ or CuSO_4_, increased the biofilm formation relative to the control medium. Exposure to H_2_O_2_ did not affect cell viability but stimulated biofilm formation. However, the same results were not replicated in the fixed-bed bioreactor, probably reflecting conditions that are unique to this environment such as the controlled pH and more vigorous aeration. Although we were able to cultivate *P. limnophila* in a fixed-bed bioreactor using a chemically defined medium, the factors that stimulate biofilm formation and inhibit planktonic growth were only identified in microtiter plates and further evaluation is required to establish optimal growth conditions in the bioreactor system.

## 1. Introduction

Planctomycetes are ubiquitous bacteria that have attracted scientific interest because their genomes contain numerous biosynthetic gene clusters [1,2,3,4,5]. They belong to the Planctomycetes-Verrucomicrobia-Chlamydiae (PVC) superphylum [6] and play a key role in the global nitrogen cycle [7,8]. Planctomycetes lack the general divisome protein FtsZ and cell division involves polar budding or binary fission [9,10]. The freshwater model strain *Planctopirus limnophila* has a dimorphic life cycle with a motile phase that allows attachment to surfaces (or each other) with its holdfast structures followed by maturation into sessile stalked mother cells that form buds [11,12]. Planctomycetes are found in soils worldwide [13], but most known strains live in aquatic habitats [14], where they often colonize biotic surfaces, such as microalgae, macroalgae, and marine snow, to form biofilms [15,16,17,18]. Early studies suggested that Planctomycetes share certain traits with eukaryotic cells, such as a nucleus-like structure [19], an endocytosis-like uptake mechanism [20], a compartmentalized cell structure [21], or a cell wall lacking peptidoglycan [22]. Due to new analytical techniques, these assumptions have recently been challenged. It has been demonstrated that Planctomycetes do possess peptidoglycan [11] and the structures previously described as cell compartments are in fact invaginations of the periplasm [23,24]. Planctomycetal uptake of macromolecules still requires more detailed characterization, however, recent studies suggest a different mechanism than the previously suspected vesicle-mediated uptake [23]. Cell surface alterations, so-called crateriform structures that form pili-like fibers, seem to be rather involved in the uptake of large polysaccharides from the environment [23].

The gene clusters present in Planctomycetes are promising sources of new bioactive substances, several of which have already been isolated [5,25,26]. However, it is unclear how the silent gene clusters are activated. From an ecological perspective, the production of secondary metabolites by planktonic cells appears to be of little benefit, as the secreted molecules are directly diluted to inactive concentrations [5]. This issue could be overcome by the development of microenvironments such as biofilms. In many biofilm-forming microorganisms, the switch from motile to sessile growth is coupled to changes in gene expression and metabolism [27,28,29,30], including the formation of secondary metabolites [31,32]. Given their slow growth, Planctomycetes still manage to dominate biofilm communities without being outcompeted by faster-growing heterotrophs [18,33,34], suggesting mechanisms to defend their habitats against competitors [26].

Biofilms are aggregated cells embedded in a self-produced matrix of exopolymeric substances consisting mainly of polysaccharides, proteins, lipids, and extracellular DNA [35,36]. Life within a biofilm offers protection against environmental insults, such as desiccation, extreme pH, heavy metals or antibiotics [37,38,39] and allows the cell community to retain and assimilate nutrients more effectively [35,40]. Biofilm formation can be considered as an adaptive response to hostile environments, triggered and controlled by an interplay of diverse environmental cues and intercellular communication [37,41,42]. Through release of self-produced signaling molecules, cells are able to interact within the biofilm regulating both its morphology and composition [5,41,43]. This type of communication, known as quorum sensing (QS), affects gene expression in a cell density-dependent manner [44] and was recently hypothesized to occur in the planctomycete strain *Stieleria maiorica* Mal15T, which produces stieleriacines, presumably to alter biofilm community composition in its natural habitat [5]. As a step toward the production of bioactive compounds in cultured *P. limnophila*, we determined how sessile growth can be improved by promoting biofilm formation in a chemically defined medium. We screened for important factors initially in microtiter plates, testing different C:N ratios, metal ions and oxidative stress (exposure to H_2_O_2_). We quantified biofilm formation by combining MTT assays, which detect living cells, and crystal violet (CV) staining, which detects living and dead cells. Finally, we selected the most important factors affecting surface growth in microtiter plates and investigated their impact in a fixed-bed bioreactor.

## 2. Materials and Methods

### 2.1. Bacterial Strain and Media

*Planctopirus limnophila* strain DSM 3776 was obtained from the German Collection of Microorganisms and Cell Cultures (Deutsche Sammlung von Mikroorganismen und Zellkulturen, DSMZ). The cells were cultivated in the recently developed chemically defined medium D1ASO [45] comprising 10 mM sodium phosphate buffer (pH 7.5), 50 mL/L amino acid solution (ASO), 34 mL/L Hutner’s salts solution, 23.18 mM KNO_3_, 2.32 mM NH_4_Cl, 10 g/L glucose and 0.02 mg/L cyanocobalamin. To assess different C:N ratios, we changed the amount of NH_4_Cl as shown in Table 1.

To investigate the influence of metal ions, we added FeSO_4_, ZnSO_4_ or CuSO_4_ at three different concentrations (50, 100 or 500 µM) to come up with the nine variants shown in Table 2. We also investigated the effect of oxidative stress on biofilm formation by adding H_2_O_2_ to the medium, reaching final concentrations of 0.005–50 mM. All chemicals were obtained from Sigma-Aldrich (Taufkirchen, Germany), Merck (Darmstadt, Germany) or Carl Roth (Karlsruhe, Germany).

### 2.2. Cultivation of P. limnophila in Microtiter Plates

Cryopreserved *P. limnophila* cells were inoculated to an initial optical density (OD_600_) of 0.2 in 1.5 mL of each medium (pH 7.5) in 24-well plates. The plates were incubated at 28 °C for 48 h, shaking at 100 rpm in a Multitron Standard orbital shaker (Infors, Bottmingen, Switzerland). Each well was lined with a Siporax Mini Professional carrier (Sera, Heinsberg, Germany) for biofilm analysis. After carrier removal, we determined the OD_600_ of the cells remaining in the liquid phase. Prior to crystal violet biofilm staining, *P. limnophila* was cultured in 96-well plates at 28 °C for 48 h without shaking. The various media were inoculated with cryopreserved cells to an initial OD_600_ of 0.2, and 150 µL was transferred to each well.

### 2.3. Cultivation of P. limnophila in the Bioreactor

Cells were cultured at 28 °C in 2-mL fixed-bed reactors, each fitted with six Siporax carriers. Three fixed beds were connected in parallel to a conditioning vessel and the culture medium was circulated using an ISM 931 peristaltic pump (Ismatec Wertheim, Germany) at a flow rate of 4 mL/min.

We used a 0.5-L MiniBio 500 stirred-tank bioreactor (Applikon, Delft, Netherlands) with a 0.3-L working volume as the conditioning vessel. The culture was agitated at 200 rpm using two Rushton impellers and was aerated with a micro-sparger at 0.1 vvm. The bioreactor was equipped with pH, temperature, and dissolved oxygen probes, and the pH was maintained at 7.5 by adding 1 M NaOH as required. The initial OD_600_ was 0.1. Biofilm formation was stimulated by the addition of sterile FeSO_4_, CuSO_4_ or NH_4_Cl as required once the conditioning vessel had reached OD_600_ = 0.5. After 60 h, the cultivation was stopped and the medium was pumped out at a flow rate of 1 mL/min. The carriers were removed from the fixed beds and prepared for biofilm analysis.

### 2.4. MTT Assay

The cell mass in the biofilm was determined as previously described [46] with modifications. Carriers were removed from the 24-well plates after 48 h and washed twice in sterile 0.9% (*w*/*v*) NaCl to remove loose cells. They were then transferred to 24-well plates containing 1.5 mL per well of fresh D1ASO medium supplemented with 150 µL 5 g/L 3-(4,5-dimethyl-2-thiazolyl)-2,5-diphenyl-2*H*-tetrazoliumbromide (MTT) and were incubated as above. The formazan product was solubilized by transferring the contents of each well to a centrifuge tube with 4.5 mL dimethylsulfoxide (DMSO) containing 0.4 M ammonia and vortexing for 2 min. The samples were centrifuged for 3 min to separate cell residues, and 75 µL of the supernatant was transferred to a 96-well plate and the absorbance was measured at 550 nm in a Synergy HT microplate reader (Bio-Tek Instruments, Winooski, VT, USA). Sterile D1ASO medium was used as the blank and was handled as described for the other samples.

The overgrown carriers from the 2-mL fixed beds were transferred to centrifuge tubes containing 6 mL fresh D1ASO medium and 0.6 mL MTT solution and were incubated for 30 min. The formazan product was solubilized by adding 18 mL ammonia containing DMSO, and subsequent steps were carried out as described above. The cell dry weight (CDW) was calculated by multiplying the absorbance reading by 3 to reflect the increase in volume from 2 mL (per fixed bed) to 6 mL.

Absorbance values were transformed to OD_600_ values based on the experimentally determined correlation between A_550nm_ and OD_600_, as shown in Equation (1) (2 h MTT assay) and Equation (2) (0.5 h MTT assay). This was derived by plotting OD_600_ against absorbance for a dilution series, followed by linear fitting at adjusted R^2^ = 0.98315 (1) and adjusted R^2^ = 0.98248 (2). The dilutions were prepared in triplicate. The relationship between OD_600_ and CDW (g/L) for *P. limnophila* is described by Equation (3) [45].

(1) $$A_{550\mathrm{nm}}=0.36452\;\cdot \;{\mathrm{OD}}_{600}+0.05059$$

(2) $$A_{550\mathrm{nm}}=0.07233\;\cdot \;{\mathrm{OD}}_{600}+0.06081$$

(3) $$\mathrm{CDW}=0.2905\;\cdot \;{\mathrm{OD}}_{600}+0.0294$$

### 2.5. Crystal Violet Staining

The total biofilm, comprising living and dead cells as well as exopolymeric substances, was quantified as previously described [47], with modifications. After the cultivation of cells in 96-well microtiter plates, the medium was discarded and the plates were washed twice with tap water to remove loose cells. The biofilms were then stained with 200 µL 0.1% (*w*/*v*) crystal violet for 15 min at room temperature. The stain was discarded and the plates were washed twice with tap water and dried at 60 °C for 3 h. The bound crystal violet was dissolved in 250 µL 70% ethanol and 150 µL of each sample was transferred to a new well. The absorbance was measured at 590 nm in the Synergy HT microplate reader.

### 2.6. Measurement of OD_600_

OD_600_ values were measured using a BioSpectrometer basic (Eppendorf, Hamburg, Germany). For readings > 0.3, samples were diluted in 0.9% (*w*/*v*) NaCl.

## 3. Results

### 3.1. Effect of C:N Ratio on Biofilm Formation in Microtiter Plates

**Figure 1:**
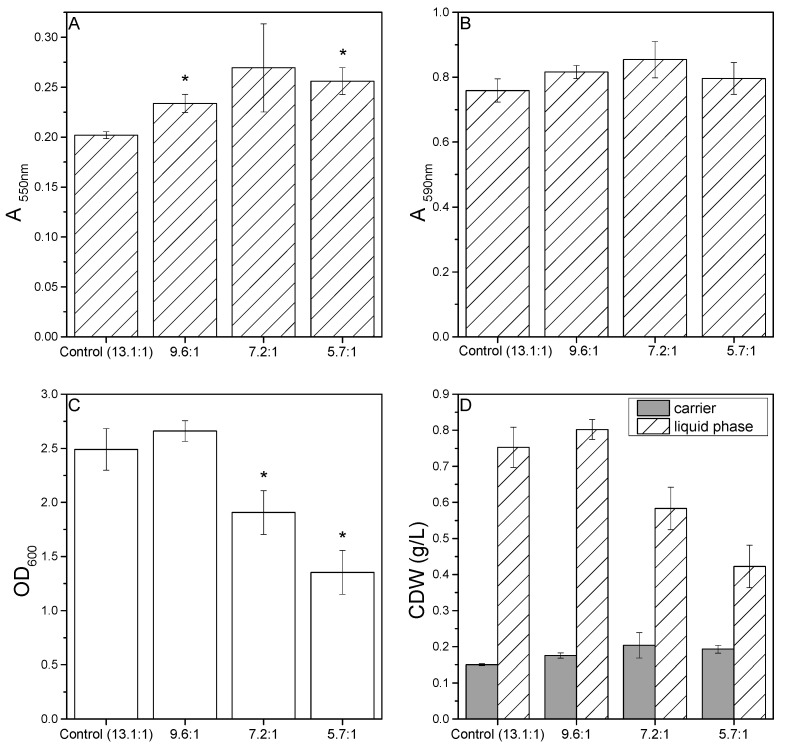
Effects of different C:N ratios on *P. limnophila* growth and biofilm formation in microtiter plates. (**A**) MTT assay results (absorbance readings at 550 nm). (**B**) Crystal violet absorbance readings at 590 nm. (**C**) OD_600_ readings in the liquid phase. (**D**) Distribution of the CDW (g/L) between the carriers and liquid phase. Data are means ± standard deviations (*n* = 3 biological replicates). Statistical analysis for MTT assay, OD_600_ and CV staining was based on a two-sample *t*-test compared to control values (D1ASO), * *P* < 0.05.

The C:N ratio of the standard D1ASO medium was 13.1:1. Reducing this ratio by adding NH_4_Cl (Table 1) had a positive effect on the sessile growth of *P. limnophila*, with more extensive carrier colonization observed in all three media variants. When biofilm formation was measured using the MTT assay, there were significant differences between the control medium and the C:N ratios of 5.7:1 and 9.6:1 (Figure 1A). The same trend was apparent when the biofilms were stained with crystal violet, although the differences were not significant (Figure 1B). The C:N ratio of 9.6:1 also resulted in a higher OD_600_ than the control medium, suggesting that the extensive colonization of the carriers was facilitated by the presence of more suspended cells that were available for surface attachment. However, a further reduction in the C:N ratio was associated with fewer cells in the liquid phase compared to the control medium, suggesting that the proliferation of planktonic cells was inhibited (Figure 1C). To visualize the biomass distribution, we converted the MTT assay and OD_600_ results to CDW concentrations using empirical correlations (Figure 1D). C:N ratios lower than 9.6:1 clearly influenced the distribution of cells between the liquid phase and carrier. This suggests that the addition of supplementary nitrogen above a threshold concentration promotes cell attachment to surfaces and fewer cells are therefore present in the liquid phase, which is consistent with previous work [48].

### 3.2. Effect of Metal Ions on Biofilm Formation in Microtiter Plates

**Figure 2:**
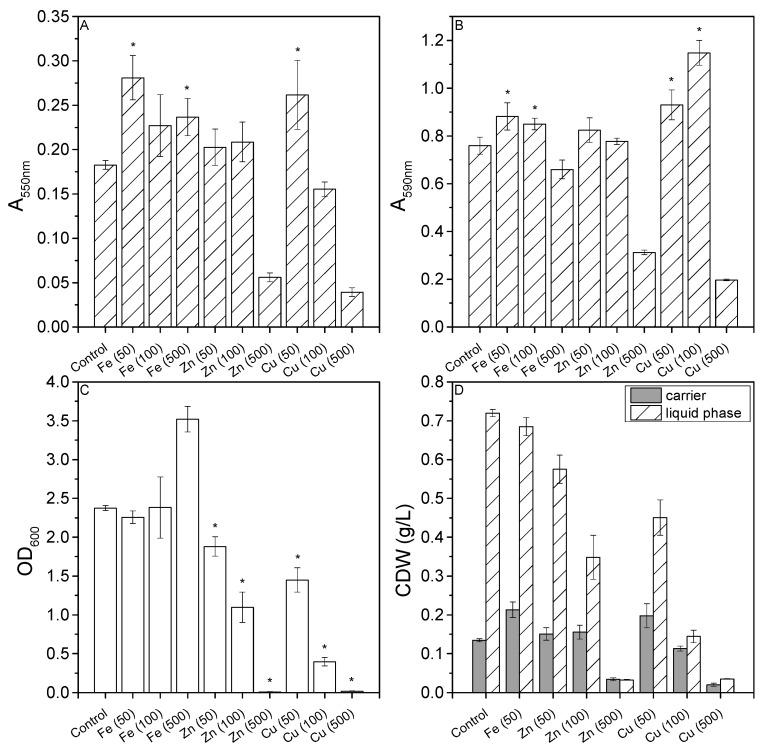
Effects of different Fe, Cu, and Zn concentrations (Table 2) on *P. limnophila* growth and biofilm formation in microtiter plates. (**A**) MTT assay results (absorbance readings at 550 nm). (**B**) Crystal violet absorbance readings at 590 nm. (**C**) OD_600_ readings in the liquid phase. (**D**) Distribution of the CDW (g/L) between the carriers and liquid phase. Data are means ± standard deviations (*n* = 3 biological replicates). Statistical analysis for MTT assay, OD_600_ and CV staining was based on a two-sample *t*-test compared to control values (D1ASO medium), * *P* < 0.05.

The metal ion content of the medium was increased by adding three different concentrations of Fe, Cu, and Zn. MTT assays indicated a significant increase in absorbance for the media supplemented with Fe (50), Fe (500), and Cu (50), indicating that these concentrations enhanced surface colonization (Figure 2A). Crystal violet staining confirmed the results for Fe (50) and Cu (50), whereas Fe (500) instead showed a decline in absorbance (Figure 2B). However, crystal violet staining revealed a significant increase in absorbance for Fe (100) and Cu (100), the former also showing an increase in the MTT assay but the latter showing a decrease in the MTT assay. The lowest absorbance values in both assays were observed when the medium was supplemented with Cu (500) and Zn (500).

Increasing the concentration of Cu or Zn universally reduced the OD_600_ in the liquid phase (Figure 2C). The OD_600_ results for Fe (100 and 500) cannot be taken at face value because Fe precipitates at concentrations exceeding 100 µM and the particles contribute to the reading. Cu (500) and Zn (500) had a bacteriostatic effect, suggesting these concentrations are toxic but sublethal.

The addition of metal ions clearly influenced the distribution of biomass between the carriers and the medium (Figure 2D). Zn (500), Cu (100), and Cu (500) resulted in a balanced ratio of sessile and motile cells but low biomass yields.

### 3.3. Effect of Oxidative Stress on Biofilm Formation in Microtiter Plates

**Figure 3:**
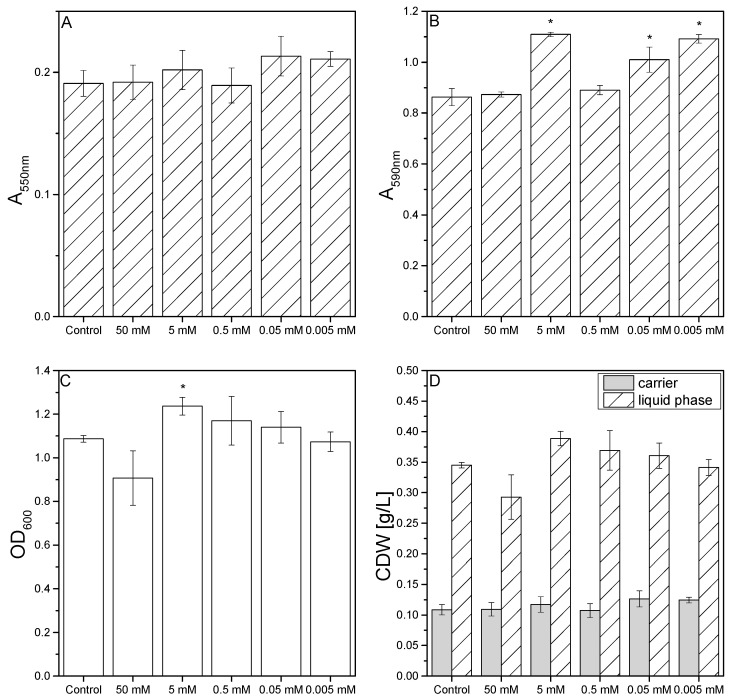
Effects of different H_2_O_2_ concentrations (5 µM to 50 mM) on *P. limnophila* growth and biofilm formation in microtiter plates. (**A**) MTT assay results (absorbance readings at 550 nm). (**B**) Crystal violet absorbance readings at 590 nm. (**C**) OD_600_ readings in the liquid phase. (**D**) Distribution of the CDW (g/L) between the carriers and liquid phase. Data are means ± standard deviations (*n* = 3 biological replicates). Statistical analysis for MTT assay, OD_600_ and CV staining was based on a two-sample *t*-test compared to control values (D1ASO medium), * *P* < 0.05.

We investigated the influence of different concentrations of H_2_O_2_ on *P. limnophila* biofilm formation because this chemical is known to trigger oxidative stress pathways in other bacteria [49]. The MTT assay indicated that H_2_O_2_ had no significant effect at concentrations between 5 µM and 50 mM (Figure 3A). In contrast, crystal violet staining showed a significant increase in biofilm formation at H_2_O_2_ concentrations of 5 mM, 50 µM, and 5 µM (Figure 3B). The OD_600_ of the liquid phase was reduced in the presence of 50 mM H_2_O_2_ indicating that cell growth was inhibited, but the OD_600_ increased significantly in the presence of 5 mM H_2_O_2_ (Figure 3C). Accordingly, the distribution of biomass between the carrier and the liquid phase was only affected at H_2_O_2_ concentrations of 5–50 mM (Figure 3D).

### 3.4. Effect of Cultivation Parameters on Biofilm Formation in Bioreactors

**Figure 4:**
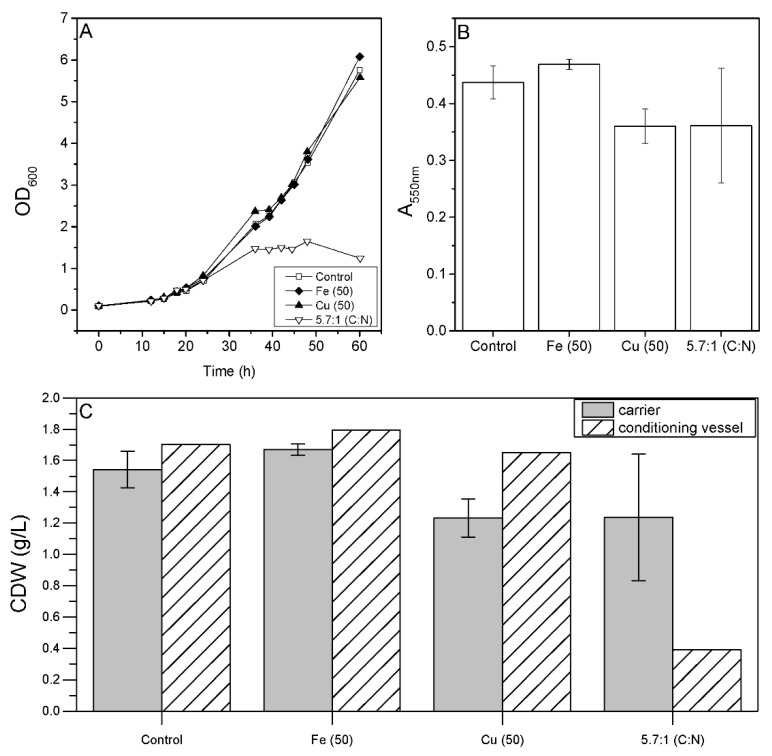
Effects 50 µM Fe or Cu, or a low C:N ratio (5.7:1) on *P. limnophila* growth and biofilm formation in a fixed-bed bioreactor. (**A**) OD_600_ in the liquid phase (conditioning vessel). (**B**) MTT assay results (absorbance readings at 550 nm). (**C**) Distribution of the CDW (g/L) between the carriers and liquid phase. Data in (**B**,**C**) are means ± standard deviations (*n* = 3 biological replicates). Statistical analysis for MTT assay was based on a two-sample *t*-test compared to control values (D1ASO medium).

Having identified the factors that affect *P. limnophila* growth and biofilm formation in microtiter plates, we investigated their impact in a fixed-bed cultivation process. OD_600_ measurements in the conditioning vessel revealed growth inhibition when the C:N ratio was reduced to 5.7:1 (Figure 4A), which is consistent with the microtiter plate screening results. However, unlike the screening experiments, we observed no significant effect when we added Fe or Cu (Figure 4A), even though the latter inhibited cell growth in microtiter plates. MTT assay revealed no significant changes compared to the control medium when we reduced the C:N ratio to 5.7:1 or increased Fe or Cu concentrations by 50 µM, although the addition of Fe resulted in a slight increase in biomass on the carriers and the lower C:N ratio, and the Cu treatment resulted in a slight reduction in biomass (Figure 4B). The distribution of biomass between the carrier and liquid phase shifted towards growth in the fixed-bed setting compared to the microtiter plates. The lower C:N ratio of 5.7:1 led to a higher biomass concentration in the fixed bed than in the conditioning vessel (Figure 4C).

## 4. Discussion

The formation of bacterial biofilms is accompanied by changes in gene expression and metabolic profiles [27,28,29], potentially involving the activation of multiple biosynthetic gene clusters. Silent biosynthetic gene clusters identified in Planctomycetes may therefore encode the enzymes responsible for the synthesis of novel bioactive compounds, but this has not been explored in detail because Planctomycetes are difficult to cultivate. The development of the chemically defined medium D1ASO overcame this hurdle for the freshwater strain *P. limnophila*, leading to CDWs exceeding 13 g/L in bioreactor processes [45].

As a step toward the production of novel secondary metabolites using *P. limnophila*, we investigated the conditions required to stimulate biofilm formation in fixed-bed bioreactors. Little is known about planctomycetal biofilm formation, so we initially focused on the identification of factors that influence growth and biofilm formation in our chemically defined medium at the microtiter plate scale. We analyzed the biofilms using two methods based on different principles: the MTT assay, in which a soluble dye is converted into an insoluble formazan product whose abundance correlates with the metabolically active biomass, and crystal violet staining, which measures the abundance of living and dead cells. The use of two methods prevents false positives in the MTT assay caused by the increased metabolic activity of cells on the carriers. The MTT assay data were prioritized for validation of the most important parameters in the bioreactors because the fixed bed should primarily contain living cells.

The microtiter plate screen with different C:N ratios revealed that higher nitrogen levels promoted sessile growth on the carriers and fewer cells accumulated in the liquid phase. Similarly, higher ammonium levels promoted sessile growth and biofilm formation by *Rhodopirellula baltica*, possibly via an Amt transporter fused to a sensory histidine kinase [48]. Genes that may encode such an ammonium sensor kinase are present in the *R. baltica* genome and in the genomes of other Planctomycetes, including *P. limnophila* [48]. The aggregation of biofilm-forming cells in response to ammonium may be a defensive reaction that reduces the surface area and creates an additional barrier to the environment [48]. Alternatively, the sudden availability of nitrogen in a nutrient-depleted environment may activate biofilm formation as a means to accumulate nutrients and protect them from competitors.

The microtiter plate screen with different metal ions revealed that Zn and Cu have a strong inhibitory effect on planktonic cells in the liquid phase. The OD_600_ in the liquid phase correlated negatively with increasing metal concentrations, but this effect was less striking in the biofilm assays. Fe (500) led to much higher absorbance readings than Zn (500) and Cu (500), indicating that Fe is less toxic toward *P. limnophila* than the other metals, even if the OD_600_ reading in the liquid phase was influenced by Fe precipitates. Both Fe and Cu induced *P. limnophila* biofilm formation.

In earlier studies, the growth of *Gemmata* spp. was enhanced by the addition of FeSO_4_ [50] and two planctomycetal strains isolated from Fe(OH)_2_ deposits were found to be attached to Fe precipitates [51]. Fe promotes biofilm formation in *Pseudomonas aeruginosa* [52,53], *Escherichia coli* [54], *Bacillus subtilis* [55], *Staphylococcus aureus* [56], and *Vibrio cholerae* [57]. In some *Campylobacter jejuni* strains, oxidative stress induced by Fe resulted in the production of more extracellular DNA and exopolymeric substances [58]. Tests with a range of metals showed that Zn was the least toxic toward *Rhodopirellula* sp. LF2, with no visible effects up to a concentration of 58.7 µM and cells remaining viable up to a concentration of 293.3 µM, probably reflecting the involvement of Zn in more physiological processes than the other metals tested [59]. Similarly, Zn at equivalent concentrations did not show negative effects against *P. limnophila* in our MTT assay, but we observed a lower OD_600_ in the liquid phase compared to the control medium. It is not possible to compare this outcome directly with the earlier study because the latter relied on the use of agar plates; hence, the distribution between sessile and motile cells was not reported [59]. The analysis of *Rhodopirellula* sp. LF2 revealed that Cu is more toxic than Zn at the same concentration [59], which also appeared to be the case for *P. limnophila* based on our OD_600_ measurements in the liquid phase. However, the MTT assay for Cu (50) and the crystal violet staining for Cu (50) and Cu (100) generated higher absorbance values compared to Zn, indicating a stronger positive effect on sessile growth. The stabilizing effect of cationic metal ions, such as Cu, Zn, and Fe, on *B. subtilis* biofilms has been previously demonstrated [55]. Therefore, further studies are required to determine whether *P. limnophila* biofilm formation is directly influenced by metal ions or whether they exert an indirect stabilizing effect.

Biofilm formation has been linked to oxidative stress in *Helicobacter influenzae* [60], *C. jejuni* [58], *Streptococcus mutans* [61], and *E. coli* [62]. H_2_O_2_ is often used to induce oxidative stress, and this was shown to trigger biofilm formation in *Mycobacterium avium* [49]. However, H_2_O_2_ had no effect against *P. limnophila* in our MTT assays although the addition of 50 mM H_2_O_2_ inhibited cell growth based on the lower OD_600_ reading in the liquid phase. Crystal violet staining indicated a significant increase in biofilm formation in the presence of 5 mM, 50 µM, and 5 µM H_2_O_2_ based on the detection of dead as well as metabolically active cells.

Having established the parameters that affected cell growth and biofilm formation in microtiter plates, we transferred the experiments to a bioreactor to evaluate the impact of such factors on a larger scale. We replicated the effect of the lower C:N ratio on motile cell growth, but not the effect of 50 µM Cu. Indeed, none of the factors identified at the microtiter plate scale significantly influenced biofilm formation in the fixed-bed reactor, and only the addition of Fe had a slight positive effect on biofilm formation. This discrepancy may reflect differences between the cultivation systems in terms of pH control and aeration. The pH was maintained at 7.5 in the bioreactor and the cells were aerated with a micro-sparger, whereas oxygen exchange in the microtiter plates occurred only by surface aeration and the pH was not regulated. Given that Fe^2+^ is oxidized to the less bioavailable Fe^3+^ at pH > 5 [50], the adjusted pH in the bioreactor may limit Fe availability, and the more intense aeration in the bioreactor may enhance the oxidation of Fe^2+^. The investigation of *Gemmata* spp. has revealed the absence of a complete set of genes involved in Fe acquisition and that growth can be enhanced by *E. coli* filtrates containing siderophores [50,63]. The ability of *P. limnophila* to take up Fe^3+^ should be evaluated in future studies.

## 5. Conclusions

Microtiter plate screening experiments revealed several factors with the potential to support *P. limnophila* sessile growth and biofilm formation in a fixed-bed reactor. We also determined the concentrations of metals, NH_4_Cl, and H_2_O_2_ that inhibit cell growth and showed that the relative proportions of sessile cells on carriers and motile cells in the liquid phase can shift depending on the C:N ratio and metal ion concentrations. The screening results suggested that a C:N ratio of 5.7:1 as well as the presence of additional 50 µM Fe or Cu significantly increased the absorbance signal in the MTT assay with largely consistent (although not statistically significant) results in the crystal violet assay. However, none of the identified factors significantly increased biofilm formation in the bioreactor, and only the presence of additional 50 µM Fe resulted in a slight positive effect on *P. limnophila* surface growth under these conditions. Overall, our data suggest that *P. limnophila* is influenced by the bioreactor system pH and/or aeration, which will be investigated in more detail in future studies. Although the factors we identified in the microtiter plate screen did not significantly increase the biomass in the bioreactor, we have nevertheless demonstrated the first successful cultivation of *P. limnophila* in a chemically defined medium using a fixed-bed bioreactor system.

## Figures and Tables

**Table 1:** Final concentrations of KNO_3_ and NH_4_Cl to modify the C:N ratio of D1ASO medium + 10 g/L glucose.

Medium	C:N	KNO_3_ (mM)	NH_4_Cl (mM)
D1ASO (control)	13.1:1	23.18	2.32
Medium 2	9.6:1	23.18	11.6
Medium 3	7.2:1	23.18	23.2
Medium 4	5.7:1	23.18	34.8

**Table 2:** Final concentrations of metal ions in D1ASO medium.

	ZnSO_4_ (µM)	CuSO_4_ (µM)	FeSO_4_ (µM)
D1ASO	6.5	0.3	15.2
Zn (50)	56.5	0.3	15.2
Zn (100)	106.5	0.3	15.2
Zn (500)	506.5	0.3	15.2
Cu (50)	6.5	50.3	15.2
Cu (100)	6.5	100.3	15.2
Cu (500)	6.5	500.3	15.2
Fe (50)	6.5	0.3	65.2
Fe (100)	6.5	0.3	115.2
Fe (500)	6.5	0.3	515.2

## Data Availability

All relevant data are contained within this article.
